# Identification of an Essential Cytoplasmic Region of Interleukin-7 Receptor α Subunit in B-Cell Development

**DOI:** 10.3390/ijms19092522

**Published:** 2018-08-25

**Authors:** Hirotake Kasai, Taku Kuwabara, Yukihide Matsui, Koichi Nakajima, Motonari Kondo

**Affiliations:** 1 Department of Immunology, Duke University Medical Center, Durham, NC 27710, USA; hirotake.kasai@yamanashi.ac.jp; 2 Department of Molecular Immunology, Toho University School of Medicine, Tokyo 143-8540, Japan; kuwabara@med.toho-u.ac.jp (T.K.); ym04083@yahoo.co.jp (Y.M.); 3 Toho University Graduate School of Medicine, Tokyo 143-8540, Japan; 4 Department of Urology, Toho University Omori Medical Center, Tokyo 143-8541, Japan; koichin@med.toho-u.ac.jp

**Keywords:** interleukin-7, interleukin-7 receptor, B-cell development, signal transducer and activator of transcription 5

## Abstract

Interleukin-7 (IL-7) is essential for lymphocyte development. To identify the functional subdomains in the cytoplasmic tail of the IL-7 receptor (IL-7R) α chain, here, we constructed a series of IL-7Rα deletion mutants. We found that IL-7Rα-deficient hematopoietic progenitor cells (HPCs) gave rise to B cells both in vitro and in vivo when a wild-type (WT) IL-7Rα chain was introduced; however, no B cells were observed under the same conditions from IL-7Rα-deficient HPCs with introduction of the exogenous IL-7Rα subunit, which lacked the amino acid region at positions 414–441 (d414–441 mutant). Signal transducer and activator of transcription 5 (STAT5) was phosphorylated in cells with the d414–441 mutant, similar to that in WT cells, in response to IL-7 stimulation. In contrast, more truncated STAT5 (tSTAT5) was generated in cells with the d414–441 mutant than in WT cells. Additionally, the introduction of exogenous tSTAT5 blocked B lymphopoiesis but not myeloid cell development from WT HPCs in vivo. These results suggested that amino acids 414–441 in the IL-7Rα chain formed a critical subdomain necessary for the supportive roles of IL-7 in B-cell development.

## 1. Introduction

Interleukin-7 (IL-7) provides signals via the IL-7 receptor (IL-7R), which are required during many stages of lymphocyte development [1,2]. IL-7R is composed of two different receptor subunits, IL-7Rα and common γ (γ_c_) chains [3,4]. IL-7Rα is also a component of the thymic stromal lymphopoietin receptor complex, whereas γ_c_ is common to functional receptor complexes for IL-2, IL-4, IL-7, IL-9, IL-15, and IL-21 [5]. The developmental processes governed by IL-7R signaling include initiation of cell proliferation, protection from apoptotic cell death, and induction of lineage-specific events (i.e., gene rearrangement in antigen receptor loci) [6]. Mutations that interfere with IL-7R signaling cause profound immunodeficiency in both humans and mice [7,8,9], highlighting the central role IL-7 plays in lymphopoiesis.

Activation of signal transduction pathways via IL-7R begins with IL-7 binding to IL-7R complexes, followed by heterodimerization and conformational changes in IL-7Rα and γ_c_ chains [10]. Next, Janus kinase 1 (JAK1) and JAK3 interphosphorylate, inducing kinase activity in both molecules. Activated JAK1 and JAK3 phosphorylate tyrosine residues on their substrates, including other kinases, adaptor molecules, and receptor subunits [11]. Phosphorylated tyrosine residues in the cytoplasmic domains of cytokine receptors can be docking sites for downstream signaling molecules with SH2 domains, such as phosphatidylinositol 3-kinase (PI3K) and signal transducer and activator of transcription 5 (STAT5) [11].

Four tyrosine residues are present in the cytoplasmic domain of mouse IL-7Rα. Among these four tyrosine residues, the third, Y449, is necessary for STAT5 binding to IL-7Rα [12,13]. Because the introduction of a constitutively active form of STAT5 in *IL-7Rα*^−/−^ hematopoietic stem cells (HSCs) rescues impaired B-cell development [14], STAT5 is considered a critical signaling molecule for IL-7R signaling [12]. Indeed, B-cell development is completely shut down in 7RαYYFY knock-in mice, in which Y449 of IL-7Rα is substituted with phenylalanine (F449). In contrast, the number of early T-cell progenitors in 7RαYYFY knock-in mice is reduced, but the number of mature T cells in the periphery is close to normal. Therefore, other functional subdomains that play a role in the regulation of signal transduction via IL-7R should exist in the cytoplasmic tail of IL-7Rα.

In addition to the tyrosine residues, two motifs, namely Box1 and Box2, which are conserved in a number of cytokine receptors, are also recognized in the IL-7Rα chain [10,15,16]. These two subdomains are known to form binding sites of JAK family kinases. Therefore, if Box1 or Box2 is removed, the mutated cytokine receptors lack the ability to trigger signals upon binding of a cognate cytokine [10,15,16]. The acidic region, which was first identified in IL-2Rβ chain and forms a binding site for Src tyrosine kinases, such as Lck and Fyn, is also found in the IL-7Rα chain [17]. However, no other functional subdomains have been identified in the cytoplasmic tail of the IL-7Rα chain.

Therefore, in the present study, we constructed a series of IL-7Rα-deletion mutants to uncover hidden functional subdomains in the cytoplasmic tail of IL-7Rα chain. Our results provide important insights into B-cell development and IL-7R signal transduction. 

## 2. Results

### 2.1. Identification of the IL-7Rα Cytoplasmic Regions that Are Necessary for IL-7-Mediated Cell Proliferation

**Figure 1:**
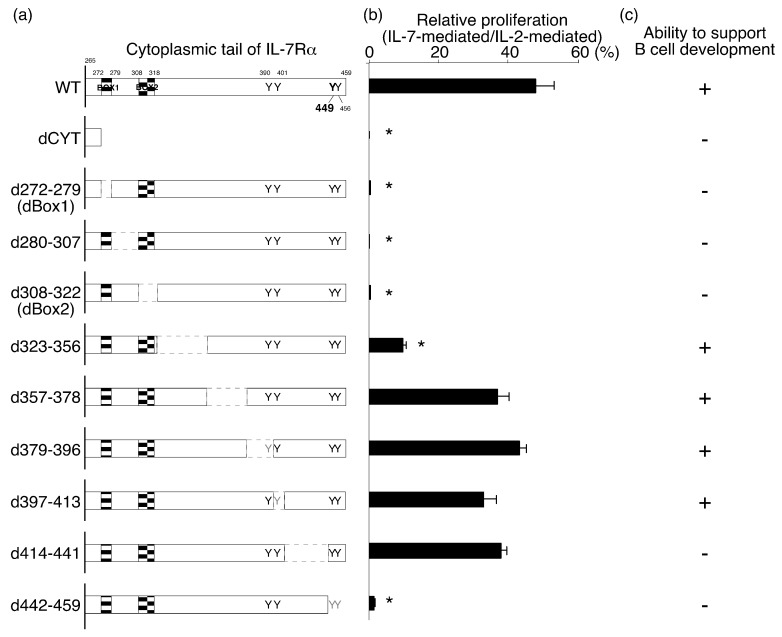
Identification of functional subdomains in the cytoplasmic tail of the IL-7Rα subunit. (**a**) Schematic structure of IL-7Rα deletion mutants. The positions of four tyrosine residues and Box1/2 in the intracytoplasmic domain of IL-7Rα are also indicated. The functionality of each mutant was tested by analysis of IL-7-mediated cell proliferation with CTLL-2 cells (**b**) and by appearance of CD19^+^ cells from IL-7Rα^−/−^ c-Kit^+^ lineage^−^ Sca-1^+^ (KLS) cells after the in vitro cultures (**c**). The data shown in (**b**) are representative of three independent experiments and normalized to the proliferation rate of each line in the presence of IL-2 to minimize variations in input cell numbers. Then, the proliferation rate of CTLL-2 cells with each mutant was compared to the rate of the cells with wild-type (WT) IL-7Rα chain (* *p* < 0.05).

We constructed a series of IL-7Rα deletion mutants to study the roles of different cytoplasmic regions in IL-7R signaling (Figure 1a). We divided the IL-7Rα cytoplasmic domain into nine segments and made deletion mutants lacking each segment. We also generated one mutant lacking all nine segments (dCyt). Two of the segments were chosen such that we could delete conserved motifs, such as Box1 (d280–307) and Box2 (d308–322) [18]. The d280–307 mutant lacked all amino acid residues between Box1 and Box2. Segments 379–396 and 397–413 were chosen so that only the first (Y390) or second (Y401) tyrosine residue was deleted (d379–396 and d397–413 mutants, respectively). The d414–441 mutant conserved all four tyrosine residues but lacked most of the amino acid residues between the second and third residues. Finally, we deleted the C terminal region of IL-7Rα, which contained the third (Y449) and fourth (Y456) tyrosine residues. 

We screened each mutant using two different criteria. The first was the ability to transduce growth signals in an IL-2-dependent mouse T-cell line (CTLL-2 cells). The other was the potential to support B-cell development from hematopoietic progenitor cells (HPCs). In this case, we purified c-Kit^+^ lineage^−^ Sca-1^+^ (KLS) HPCs from the bone marrow of *IL-7Rα*^−/−^ mice and introduced wild-type (WT) and mutant IL-7Rα chains using a retroviral system. We used the nonfunctional IL-7Rα YYFY mutant as a negative control and the WT IL-7Rα chain as a positive control. After mutant IL-7Rα chains were introduced into *IL-7Rα*^−/−^ HPCs, we cultured the *IL-7Rα*^−/−^ HPCs with exogenous WT or mutant IL-7Rα subunits on OP9 cells in the presence of stem cell factor (SCF), Flt3 ligand, and IL-7 for 6 days. Then, we examined the presence of CD19^+^ cells after culture.

As was the case with other cytokine receptor subunits, Box1 and Box2 were indispensable for IL-7Rα function based on the IL-7-mediated proliferation of CTLL-2 cells (see d272–279 and d308–322 mutants in Figure 1b). In addition, the d280–307 mutant, which lacked the region between Box1 and Box2, was not functional. Thus, dBox1, d280–307, and dBox2 mutants were not functional, perhaps due to the lack of association with JAK1 [19,20]. The d442–459 mutant was nonfunctional as well, presumably because of the lack of STAT5 binding in the absence of Y449. Although the d323–356 mutant showed lower functionality than the wild-type (WT) IL-7Rα chain, other mutants (i.e., d357–378, d379–396, d397–413, and d414–441) induced cell proliferation at a level comparable to that of the WT IL-7Rα chain (Figure 1b).

### 2.2. The Amino Acids Region from Positions 414 to 441 of the IL-7Rα Subunit Was Necessary for B-Cell Development

**Figure 2:**
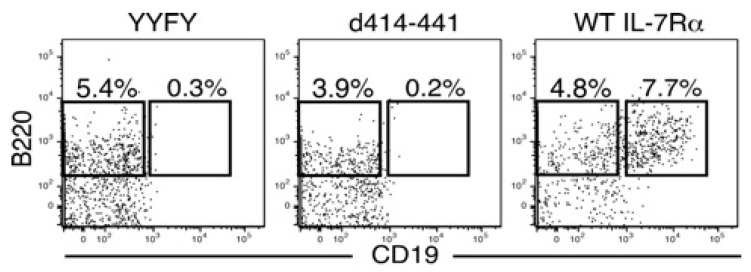
The d414–441 mutant did not support IL-7-mediated stage transition from the pre-proB (B220^+^CD19^−^) to proB stage (B220^+^CD19^+^) during B-cell development. KLS cells from *IL-7Rα*^−/−^ bone marrow were infected with retroviruses expressing the YYFY mutant (negative control), d414–441 mutant, or WT IL-7Rα (positive control). Two days after infection, green fluorescent protein (GFP)-positive cells were purified by fluorescence-assisted cell sorting (FACS) and cultured on OP9 stromal cell layers in the presence of stem cell factor (SCF), Flt3 ligand, and IL-7 for 6 days. Cells were then stained with anti-B220 and anti-CD19 antibodies and analyzed by FACS.

**Figure 3:**
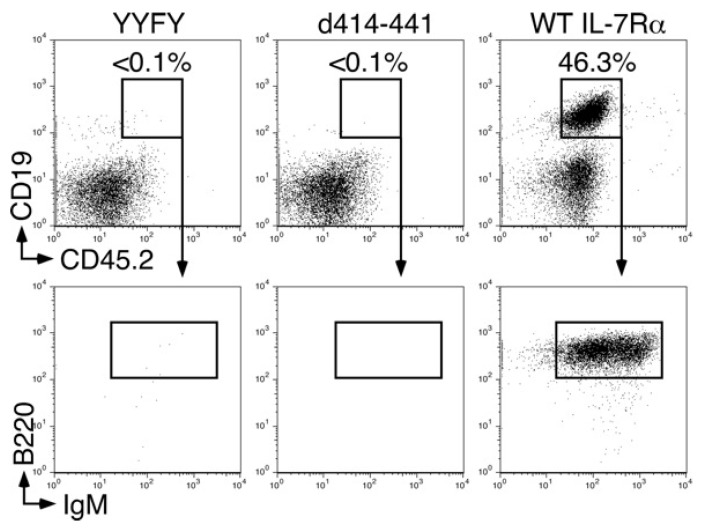
The d414–441 mutant did not support B-cell development in vivo. We infected *IL-7Rα*^−/−^ KLS cells (CD45.2) with recombinant viruses, as indicated in the figure. GFP-positive cells were purified and injected into 400 rad-irradiated *RAG2*^−/−^ (CD45.1) mice intravenously. Four weeks after injection, splenocytes from host mice were stained with anti-CD45.2, anti-CD19, anti-B220, and IgM antibodies and analyzed by FACS.

There was no discrepancy between the ability to transduce growth signals and the potential to support B-cell development except for with the d414–441 mutant (Figure 1 and Figure 2). Stagnation of B-cell development in IL-7Rα^−/−^ HPCs with the d414–441 mutant occurred at the transition stage from pre-proB (B220^+^CD19^−^) to proB (B220^+^CD19^+^) cells (Figure 2), where IL-7 stimulation was indispensable for the stage transition [14]. We also examined B-cell differentiation potential in KLS cells with the d414–441 mutant in vivo by injecting the cells into 400 rad-irradiated *recombinant activating gene 2* (*RAG2*)^−/−^ mice. We found that no B cells were derived from *IL-7Rα*^−/−^ HPCs with the d414–441 mutant, as was the case for *IL-7Rα*^−/−^ HPCs with the nonfunctional IL-7Rα YYFY mutant at 5 weeks after injection (Figure 3). These results indicated that the amino acid region from positions 414 to 441 of IL-7Rα played a critical role in IL-7-dependent B-lymphocyte development. 

### 2.3. The Truncated Form of STAT5 (tSTAT5) Was Upregulated in CTLL-2 Cells with the d414–441 Mutant Compared with that in WT Cells after IL-7 Stimulation

**Figure 4:**
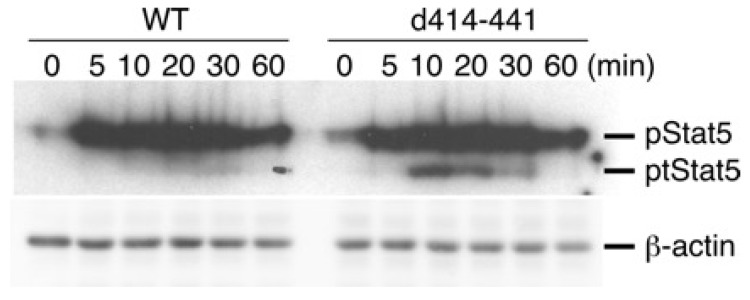
Signal transducer and activator of transcription 5 (STAT5) phosphorylation in response to IL-7 treatment in CTLL-2 cells expressing WT IL-7Rα and d414–441 mutant. Cells were starved of IL-2 for 8 h and stimulated with 100 ng/mL IL-7 for the indicated times. Whole cell lysates were subjected to sodium dodecyl sulfate polyacrylamide gel electrophoresis, and STAT5 activation was analyzed with immunoblotting using anti-phospho-STAT5 antibodies. Expression of β-actin was also examined with anti-β-actin antibodies as a loading control.

The d414–441 mutant could transduce growth signals in CTLL-2 cells as efficiently as WT IL-7Rα (Figure 1) but did not support B-lymphocyte development (Figure 2 and Figure 3). Because STAT5 plays a critical role in IL-7R signaling, we examined the phosphorylation status of STAT5 in CTLL-2 cells with the d414–441 mutant after IL-7 stimulation. As shown in Figure 4, the level of STAT5 phosphorylation in the d414–441 mutant was comparable to that in WT cells. Moreover, tSTAT5 was obviously upregulated in CTLL-2 cells with the d414–441 mutant than in cells expressing WT IL-7Rα (Figure 4). 

**Figure 5:**
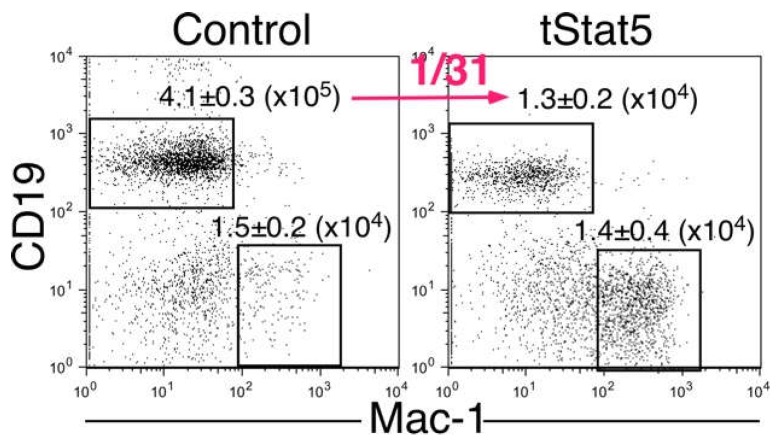
Truncated STAT5 (tSTAT5) had inhibitory functions in B-cell development. KLS cells from WT mice were infected with control or tSTAT5 retroviruses. These cells were injected into 400 rad-irradiated RAG2^−/−^ mice. Recipient-derived cells in the spleens of host mice were analyzed at 5 weeks after injection. The numbers in the plots were the means of B and myeloid cell numbers from three mice.

tSTAT5 can be generated by partial proteolysis after stimulation with cytokines, including IL-2 and IL-3. We previously demonstrated that tSTAT5 is a dominant-negative form of STAT5 [21]. Therefore, we examined whether B-cell development from HPCs was blocked in the presence of tSTAT5. For this purpose, we purified KLS cells as HPCs from WT mouse bone marrow. After the introduction of tSTAT5 (or control) in WT HPCs using a retroviral system, we injected these cells into RAG2^−/−^ mice. We then examined spleen cells in RAG2^−/−^ mice with HPCs with or without tSTAT5 at 5 weeks after injection. We found that B-cell development was severely impaired in the presence of tSTAT5, although the number of Mac-1^+^ myeloid cells was not changed (Figure 5). These results suggested that the amino acid region from positions 414 to 441 of the IL-7Rα chain may form a docking site for the molecule, which inhibits the generation of tSTAT5 in cells after IL-7 stimulation.

## 3. Discussion

**Figure 6:**
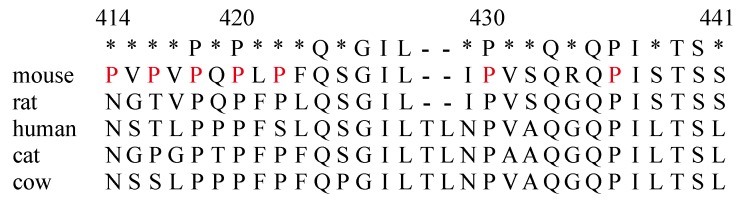
Comparison of the 414–441 region with the corresponding sites of IL-7Rα in various species. The 414-441 region contains proline residues (in red). These amino acids may provide docking site for the SH3 domain.

Activation of cytokine receptor signals is triggered by JAKs, resulting in phosphorylation of tyrosine residues of various signal molecules and the cytoplasmic tail of receptor subunits [22]. Therefore, among various protein modifications, phosphorylation is a main driver of signal cascades via cytokine receptors upon cognate ligand binding. However, other protein modifications are necessary for proper cytokine receptor signal transduction. For example, a number of molecules are acetylated in the cytoplasm, playing a role in positive regulation of interferon receptor signals [23]. Acetylated STAT1 and STAT2 exhibit enhanced transcriptional activity upon activation by tyrosine phosphorylation. Recently, we demonstrated that acetylation of JAK1, JAK3, and STAT5 occurs immediately in T cells in an IL-2-dependent manner [21]. This acetylation occurs via CREB binding protein (CBP), which relocates from the nucleus to the cytoplasm upon IL-2 stimulation. Acetylated STAT5 is a target of STAT5 protease, resulting in limited proteolysis and generation of tSTAT5. In this study, we demonstrated that tSTAT5 generation was increased after IL-7 stimulation if the 414–441 region in the IL-7Rα subunit was deleted. Therefore, we propose that the 414–441 region in the IL-7Rα subunit may form a binding site for inhibitors of CBP function. As shown in Figure 6, the 414–441 region contains multiple proline residues, which may serve as a docking site for the SH3 domain, although a conventional PxxP motif is absent. Accordingly, it is difficult to hypothesize which molecules may associate with the 414–441 region just based on the amino acid sequence. 

Various deletion mutants and point mutants of cytokine receptor subunits have been generated to identify the functional subdomains in the cytoplasmic tails of receptor subunits [24,25,26]. As a result, multiple signal pathways have been shown to be activated by different regions of the cytoplasmic tails of cytokine receptors. These studies have also shown that proliferation and differentiation signals are independent of one another. Accordingly, the d414–441 IL-7Rα mutant retained the potential to stimulate cell proliferation but lacked the ability to support B-cell development from HPCs. However, it is unclear why there was such a discrepancy despite the observation that STAT5 plays roles in both cell proliferation and support of B-cell development. In B-cell development, the first checkpoint at which IL-7 stimulation is required is the transition from the pre-proB cell population to the proB cell population. Expression of the transcription factor early B-cell factor (EBF) is indispensable for this stage transition [27]. We previously demonstrated that IL-7 stimulation is necessary for upregulation of EBF before entry to the proB cell stage [14]. Moreover, there was a threshold for EBF expression that was sufficient for the transition to the proB cell population from more immature cells. In addition, B-cell progenitors need to be stimulated with IL-7 before the pre-proB cell stage to enable sufficient EBF expression in response to IL-7 for the transition to the proB stage [28]. Therefore, one possible explanation for the discrepancy between proliferation and support of B-cell development by the d414–441 mutant may be related to the sensitivity of the mutant to the strength of STAT5 activity. Transcription levels of EBF could be more sensitive to STAT5 activity than proliferation. Furthermore, IL-7-induced EBF expression is mediated directly by STAT5, whereas cell proliferation is regulated by not only STAT5 but also other signaling components, such as PI3K, which associates with IL-7Rα at the region containing Y449 [29]. Since lack of a hypothetical molecule associated with the 414–441 region of the IL-7Rα chain may diminish IL-7R function in B-cell development, it is possible that the associated molecule plays a role in the regulation of B-cell number in vivo. Further investigations are necessary to determine why IL-7-stimulated cell proliferation and B-cell development have different requirements for the 414–441 region of IL-7Rα.

Notably, tSTAT5 inhibited IL-2-mediated proliferation of CTLL-2 cells, in contrast to IL-7-mediated cell growth. In all of our experiments, IL-2 was found to stimulate cell proliferation more strongly than IL-7; IL-7-driven cell proliferation levels which were only approximately 40% of that induced by IL-2. Therefore, cell proliferation in response to IL-2 may be more sensitive to negative effects, such as the presence of tSTAT5, than IL-7-mediated stimulation. Additionally, the effects of deletion of amino acids 414–441 from IL-7Rα may be different between T-cell development and B lymphopoiesis, as was the case in 7RαYYFY knock-in mice. Further studies using d414–441 mutant knock-in mice are needed to obtain insights into possible differential roles of the 414–441 region of IL-7Rα in T- and B-cell development. These future studies are expected to highlight the importance of acetylation in the regulation of signal transduction via cytokine receptors.

## 4. Materials and Methods

### 4.1. Mice

*IL-7Rα*^−/−^ and *RAG2*^−/−^ (CD45.1) mice on a C57Bl/6 background were bred, maintained under a specific pathogen-free environment at the Duke University Medical Center Animal Care Facility and the animal facility at Toho University School of Medicine, and used at 8–12 weeks of age. All studies and procedures were approved by the Duke University Animal Care and Use Committee (A246-07-09, 24 September 2009) and Toho University Administrative Panel for Animal Care (18-54-311, 1 April 2018) and Recombinant DNA (18-54-303, 1 April 2018).

### 4.2. Construction of Mutant IL-7Rα Subunits and Retrovirus Production

IL-7Rα deletion mutants shown in Figure 1 were generated by polymerase chain reaction (PCR) using full-length mouse IL-7Rα cDNA as a template and the following primers: 5′-tcaaggaggatgggatcc-3′ (common forward primer) and 5′-tacatagttgttccagagttttcttgacaggtttattcttttttttccat-3′ (272-279, reverse primer), 5′-cttcaacgcctttcacctcatgagtatgatcggggagactaggccatacg-3′ (280-307, reverse primer), 5′-gtgcaggaagatcattgggcagaaactggcagtccaggaaactttcggga-3′ (308-322, reverse primer), 5′-ctcttctaactgtttctggtgggctactttccacctcgtccctggcttca-3′ (323-356, reverse primer), 5′-tagaggaaaggagtggaggggcattgctgactgaagtctcaggcgagcggttt-3′ (367-378, reverse primer), 5′-agtcttgatacacaggaggcttattattgcaggtactcagatttctagcc-3′ (379-396, reverse primer), 5′-atggttgagggacagggacagggaccctatttctgtcaccatctctgtagtca-3′ (397-413, reverse primer), 5′-catacgcttcttcttgattcagtacgacatttgtgtttccagagtttggc-3′ (414-441, reverse primer), and 5′-tgaggaagtggagatgggctg-3′ (442-459, reverse primer). The IL-7Rα YYFY mutant, in which Y449 was substituted with F, was generated by site-directed mutagenesis. Mutants were confirmed by sequencing. All cDNAs were subcloned between the 5′-long terminal repeat and internal ribosome entry site (IRES) in the Murine Stem Cell Virus (MSCV)-IRES-green fluorescent protein (GFP) vector. IL-7Rα and tSTAT5 in the MSCV-IRES-GFP vector were described previously [14,21,30,31]. Retroviruses were prepared as described in [14].

### 4.3. Establishment of CTLL-2 Transfectants

CTLL-2 IL-2-dependent mouse T cells were cultured in complete medium (RPMI 1640 with 10% fetal calf serum [FCS] and 50 μM 2-mercaptoethanol) supplemented with 2 ng/mL hIL-2. CTLL-2 transfectants stably expressing WT or mutant IL-7Rα subunits were established with retroviral systems, followed by purification of GFP^+^ cells by fluorescence-assisted cell sorting (FACS) as described previously [32]. All GFP^+^ cells expressed exogenously introduced genes, as shown by staining for cell surface IL-7Rα. 

### 4.4. Proliferation Assays

CTLL-2 transfectants were maintained in complete medium supplemented with hIL-2. After washing three times with phosphate-buffered saline, 5 × 10^4^ cells in complete medium were cultured in each well of a 96-well plate in the presence of IL-7 (10 ng/mL) at 37 °C for 48 h. [^3^H]-thymidine (1 μCi) was added to the culture 4 h before harvesting. Cells were harvested with an automatic cell harvester on a glass filter (Harvester 96; TOMTEC, Hamden, CT, USA). Radioactivity was determined USA).

### 4.5. Cell Sorting and FACS Analysis

Antibodies used in FACS sorting and analyses were as follows: phycoerythrin (PE)- or biotin-conjugated anti-IL-7Rα (A7R34); PE/Cy5- or allophycocyanin (APC)-conjugated anti-B220 (RA3-6B2); PE-anti-CD19 (6D5); fluorescein isothiocyanate (FITC)-, PE-, or PE-/Cy5-conjugated anti-Mac-1 (M1/70); PE/Cy5-conjugated anti-CD3 (145-2C11); anti-CD4 (RM4-5); anti-CD8 (53-6.7); anti-Gr-1 (RB6-8C5); anti-TER119 antibodies; and APC-conjugated anti-c-Kit (2B8); all antibodies were purchased from eBioscience (San Diego, CA, USA), Tombo (San Diego, CA, USA), or BD Bioscience (Mountan View, CA, USA). Alexa Fluor 594-anti-Sca-1 antibodies were prepared in our laboratory using standard procedures. Biotin-conjugated antibodies were visualized with PE-streptavidin (eBioscience).

The HPCs used in this paper were KLS cells, in which HSCs were highly enriched [33,34]. For cell surface phenotyping, cells were incubated with normal rat IgG (Sigma, St. Louis, MO, USA) and then fluorescence- or biotin-conjugated antibodies on ice for 20 min. If necessary, cells were further incubated with PE-streptavidin after washing with staining medium (Hanks’ Balanced Salt solution (HBSS) with 2% FCS and 0.02% NaN_3_). FACS analysis was performed using a FACSVantage with the DiVa option equipped with 488-nm argon, 599-nm dye, and 408-nm krypton lasers (BD Bioscience Flow Cytometry Systems) at the FACS facility of Duke University Comprehensive Cancer Center. A FACSAriaIII at the FACS facility of Toho University School of Medicine was also used for cell sorting. In addition, an LSRFortessa X-20 (BD Bioscience) was used for analyses. Data were analyzed with FlowJo software (BD Bioscience). Dead cells were excluded from analyses and sorting as cells showing positive staining with propidium iodide or 7-AAD. 

### 4.6. Immunoblotting 

CTLL-2 and its derivatives were cultured in complete medium without IL-2 for 6 h. These factor-starved cells were stimulated with a saturating amount of IL-7 (100 ng/mL) for the indicated times (Figure 4) at 37 °C. Cells were centrifuged and solubilized with lysis buffer (1% Triton X-100, 50 mM Tris-Cl, 300 mM NaCl, and 5 mM ethylenediaminetetraacetic acid) with protease inhibitor cocktail and phosphatase inhibitor cocktail (Sigma). After centrifugation, cell lysates were subjected to sodium dodecyl sulfate polyacrylamide gel electrophoresis and electrophoretically transferred to Immobilon-FL membranes (Millipore, Burlington, MA, USA). After blocking with 3% bovine serum albumin (BSA) in TBS-T (10 mM Tris-HCl, 150 mM NaCl, 0.1% Tween 20, pH = 7.5), membranes were incubated with anti-phospho-STAT5 (Tyr694) antibodies (Cell Signaling Technology, Danvers, MA, USA) in 1% BSA in TBS-T. After washing, membranes were further incubated with Alexa Fluor 680-conjugated anti-rabbit immunoglobulin (Molecular Probes, Eugene, OR, USA). Membranes were analyzed using an Odyssey infrared imaging system (LI-COR, Lincoln, NE, USA).

### 4.7. In Vitro and In Vivo Differentiation Assays 

In vitro culture of HPCs was performed as described previously [28]. In brief, after retroviral infection, HPCs were cultured on OP9 cells in the presence of IL-7, Flt3 ligand, and SCF for 6 days. In vivo injections were performed as described in Reference [28] as well. In the experiment shown in Figure 3, HPCs were purified from *IL-7Rα*^−/−^ mice (CD45.2) and were intravenously injected into 400 rad-irradiated *RAG2*^−/−^ (CD45.1) mice. For the investigation shown in Figure 5, HPCs were purified from C57Bl/6 mice (CD45.2) and intravenously injected into 400 rad-irradiated *RAG2*^−/−^ mice (CD45.1).

## Abbreviations

CBP	CREB-binding protein
CD	Cluster of differentiation
EBF	Early B cell factor
ETP	Early T cell progenitor
γ_c_	Common γ
HPC	Hematopoietic progenitor cell
Lck	Lymphocyte-specific protein tyrosine kinase
IL	Interleukin
JAK	Janus kinase
KLS	c-Kit^+^ Lineage^−^ Sca-1^+^
RAG	Recombination activating gene
Src	Rat sarcoma
SCF	Stem cell factor
STAT	Signal transducer and activator of transcription

## References

[1] Hofmeister R., Khaled A.R., Benbernou N., Rajnavolgyi E., Muegge K., Durum S.K. (1999). Interleukin-7: Physiological roles and mechanisms of action. Cytokine Growth Factor Rev..

[2] Ceredig R., Rolink A.G. (2012). The key role of IL-7 in lymphopoiesis. Semin. Immunol..

[3] Sugamura K., Asao H., Kondo M., Tanaka N., Ishii N., Ohbo K., Nakamura M., Takeshita T. (1996). The interleukin-2 receptor gamma chain: Its role in the multiple cytokine receptor complexes and T cell development in XSCID. Annu. Rev. Immunol..

[4] Leonard W.J. (1996). The molecular basis of X-linked severe combined immunodeficiency: Defective cytokine receptor signaling. Annu. Rev. Med..

[5] Rochman Y., Spolski R., Leonard W.J. (2009). New insights into the regulation of T cells by γ_c_ family cytokines. Nat. Rev. Immunol..

[6] Corfe S.A., Paige C.J. (2012). The many roles of IL-7 in B cell development; mediator of survival, proliferation and differentiation. Semin. Immunol..

[7] Puel A., Ziegler S.F., Buckley R.H., Leonard W.J. (1998). Defective IL7R expression in T^−^B^+^NK^+^ severe combined immunodeficiency. Nat. Genet..

[8] Peschon J.J., Morrissey P.J., Grabstein K.H., Ramsdell F.J., Maraskovsky E., Gliniak B.C., Park L.S., Ziegler S.F., Williams D.E., Ware C.B. (1994). Early lymphocyte expansion is severely impaired in interleukin 7 receptor-deficient mice. J. Exp. Med..

[9] Von Freeden-Jeffry U., Vieira P., Lucian L.A., McNeil T., Burdach S.E., Murray R. (1995). Lymphopenia in interleukin (IL)-7 gene-deleted mice identifies IL-7 as a nonredundant cytokine. J. Exp. Med..

[10] Kittipatarin C., Khaled A.R. (2007). Interlinking interleukin-7. Cytokine.

[11] Leonard W.J. (2001). Role of Jak kinases and STATs in cytokine signal transduction. Int. J. Hematol..

[12] Jiang Q., Benbernou N., Chertov O., Khaled A.R., Wooters J., Durum S.K. (2004). IL-7 induces tyrosine phosphorylation of clathrin heavy chain. Cell Signal.

[13] Lin J.X., Migone T.S., Tsang M., Friedmann M., Weatherbee J.A., Zhou L., Yamauchi A., Bloom E.T., Mietz J., John S. (1995). The role of shared receptor motifs and common Stat proteins in the generation of cytokine pleiotropy and redundancy by IL-2, IL-4, IL-7, IL-13, and IL-15. Immunity.

[14] Kikuchi K., Lai A.Y., Hsu C.L., Kondo M. (2005). IL-7 receptor signaling is necessary for stage transition in adult B cell development through up-regulation of EBF. J. Exp. Med..

[15] Tanner J.W., Chen W., Young R.L., Longmore G.D., Shaw A.S. (1995). The conserved box 1 motif of cytokine receptors is required for association with JAK kinases. J. Biol. Chem..

[16] Jiang Q., Li W.Q., Hofmeister R.R., Young H.A., Hodge D.R., Keller J.R., Khaled A.R., Durum S.K. (2004). Distinct regions of the interleukin-7 receptor regulate different Bcl2 family members. Mol. Cell Biol..

[17] Page T.H., Lali F.V., Foxwell B.M. (1995). Interleukin-7 activates p56*^lck^* and p59*^fyn^*, two tyrosine kinases associated with the p90 interleukin-7 receptor in primary human T cells. Eur. J. Immunol..

[18] Murakami M., Narazaki M., Hibi M., Yawata H., Yasukawa K., Hamaguchi M., Taga T., Kishimoto T. (1991). Critical cytoplasmic region of the interleukin 6 signal transducer gp130 is conserved in the cytokine receptor family. Proc. Natl. Acad. Sci. USA.

[19] Zhu M.H., Berry J.A., Russell S.M., Leonard W.J. (1998). Delineation of the regions of interleukin-2 (IL-2) receptor β chain important for association of Jak1 and Jak3. Jak1-independent functional recruitment of Jak3 to Il-2Rβ. J. Biol. Chem..

[20] Palmer M.J., Mahajan V.S., Trajman L.C., Irvine D.J., Lauffenburger D.A., Chen J. (2008). Interleukin-7 receptor signaling network: An integrated systems perspective. Cell Mol. Immunol..

[21] Kuwabara T., Kasai H., Kondo M. (2016). Acetylation Modulates IL-2 Receptor Signaling in T. Cells. J. Immunol..

[22] Leonard W.J., O’Shea J.J. (1998). Jaks and STATs: Biological implications. Annu. Rev. Immunol..

[23] Tang X., Gao J.S., Guan Y.J., McLane K.E., Yuan Z.L., Ramratnam B., Chin Y.E. (2007). Acetylation-dependent signal transduction for type I interferon receptor. Cell.

[24] Ishihara K., Hirano T. (2002). Molecular basis of the cell specificity of cytokine action. Biochim. Biophys. Acta.

[25] Minami Y., Taniguchi T. (1995). IL-2 signaling: Recruitment and activation of multiple protein tyrosine kinases by the components of the IL-2 receptor. Curr. Opin. Cell Biol..

[26] Okuda K., Foster R., Griffin J.D. (1999). Signaling domains of the βc chain of the GM-CSF/IL-3/IL-5 receptor. Ann. N. Y. Acad. Sci..

[27] Lin H., Grosschedl R. (1995). Failure of B-cell differentiation in mice lacking the transcription factor EBF. Nature.

[28] Kikuchi K., Kasai H., Watanabe A., Lai A.Y., Kondo M. (2008). IL-7 specifies B cell fate at the common lymphoid progenitor to pre-proB transition stage by maintaining early B cell factor expression. J. Immunol..

[29] Rothenberg E.V. (2014). Transcriptional control of early T and B cell developmental choices. Annu. Rev. Immunol..

[30] Kondo M., Scherer D.C., Miyamoto T., King A.G., Akashi K., Sugamura K., Weissman I.L. (2000). Cell-fate conversion of lymphoid-committed progenitors by instructive actions of cytokines. Nature.

[31] Hsu C.L., King-Fleischman A.G., Lai A.Y., Matsumoto Y., Weissman I.L., Kondo M. (2006). Antagonistic effect of CCAAT enhancer-binding protein-α and Pax5 in myeloid or lymphoid lineage choice in common lymphoid progenitors. Proc. Natl. Acad. Sci. USA.

[32] Hsu C.L., Kikuchi K., Kondo M. (2007). Activation of mitogen-activated protein kinase kinase (MEK)/extracellular signal regulated kinase (ERK) signaling pathway is involved in myeloid lineage commitment. Blood.

[33] Adolfsson J., Mansson R., Buza-Vidas N., Hultquist A., Liuba K., Jensen C.T., Bryder D., Yang L., Borge O.J., Thoren L.A. (2005). Identification of Flt3^+^ lympho-myeloid stem cells lacking erythro-megakaryocytic potential a revised road map for adult blood lineage commitment. Cell.

[34] Christensen J.L., Weissman I.L. (2001). Flk-2 is a marker in hematopoietic stem cell differentiation: A simple method to isolate long-term stem cells. Proc. Natl. Acad. Sci. USA.

